# Fluorescence-based Sensing of 2,4,6-Trinitrotoluene (TNT) Using a Multi-channeled Poly(methyl methacrylate) (PMMA) Microimmunosensor

**DOI:** 10.3390/s100100876

**Published:** 2010-01-22

**Authors:** Paul T. Charles, Andre A. Adams, Peter B. Howell, Scott A. Trammell, Jeffrey R. Deschamps, Anne W. Kusterbeck

**Affiliations:** Center for Bio/Molecular Science and Engineering (Code 6920), US Naval Research Laboratory, 4555 Overlook Ave. SW, Washington, DC 20375, USA; E-Mails: andre.adams@nrl.navy.mil (A.A.A.); peter.howell@nrl.navy.mil(P.B.H.); scott.trammell@nrl.navy.mil (S.A.T.); jeff.deschamps@nrl.navy.mil (J.R.D.); anne.kusterbeck@nrl.navy.mil (A.W.K.)

**Keywords:** TNT, immunosensor, fluorescence, PMMA, microchannel, antibody

## Abstract

Fluorescence immunoassays employing monoclonal antibodies directed against the explosive 2,4,6-trinitrotoluene (TNT) were conducted in a multi-channel microimmunosensor. The multi-channel microimmunosensor was prepared in poly (methyl methacrylate) (PMMA) *via* hot embossing from a brass molding tool. The multi-channeled microfluidic device was sol-gel coated to generate a siloxane surface that provided a scaffold for antibody immobilization. AlexaFluor-cadaverine-trinitrobenzene (AlexaFluor-Cad-TNB) was used as the reporter molecule in a displacement immunoassay. The limit of detection was 1–10 ng/mL (ppb) with a linear dynamic range that covered three orders of magnitude. In addition, antibody crossreactivity was investigated using hexahydro-1,3,5-triazine (RDX), HMX, 2,4-dinitrotoluene (DNT), 4-nitrotoluene (4-NT) and 2-amino-4,6-DNT.

## 1. Introduction

Characterization and remediation of military sites contaminated with energetic materials has continued to be an important topic in recent years. Two of the three major classes of energetic materials are nitroaromatics and nitramines [e.g., 2,4,6-trinitrotoluene (TNT) and hexahydro-1,3,5-triazine (RDX)]. These materials are known to penetrate the soil and dissolve into the groundwater or seawater with the potential for endangering the marine environment [1]. TNT also has the capability of being absorbed through the skin in humans and other living species resulting in acute and/or chronic reactions [2]. Based on EPA evaluations TNT has been classified as a “possible human carcinogen”. These health risks to humans along with the toxic and mutagenic effects to all other life forms has generated a sense of urgency to develop new technologies that can detect, contain and remediate these toxic substances. With TNT toxic levels reported at 2.0 ng/mL by the Environmental Protection Agency (EPA) in both soil and groundwater [3], new methods and devices must have the capability of detecting explosive concentrations at trace levels, but also must be designed for field deployment to enable on-site analysis. Deployment of such novel devices will not only help to monitor the movement of these energetic materials as they migrate in underground plumes, but will significantly aid in the remediation efforts by reducing costs associated with sampling and analysis.

Sensors to detect explosives have been designed and engineered in a number of formats [4–6]. Most prominent are the electrochemical sensors that employ square wave voltammetry. Wang and colleagues recently developed a flow through device for the detection of TNT. Using a carbon fiber working microelectrode employing a reduction process of TNT they were able to demonstrate detection at 100 ppb for environmental samples [7–9]. The development of a capillary electrophoresis microchip for TNT detection in non-aqueous media followed [10]. Combining a pre-concentration step (solid phase extraction matrix) with an electrophoresis microchip improved detection to sub parts-per-billion levels. Trammell and colleagues also demonstrated that TNT could be detected using interdigitated array (IDAs) gold electrodes [11]. Using an amplified redox cycling method at the IDAs surface TNT could be detected at concentrations of 6 ng/mL (ppb) with a linear response from 10–10,000 ng/mL. Although low ppb detection was achieved, the kinetics involved in the electrochemical transformations at the electrode surface was reported as a potential limiting factor.

Technologies that use biomolecules to detect TNT have also been employed. Surface plasmon resonance (SPR) sensors are one of the many techniques that rely on changes in resonance angles when biomolecular interactions occur between an immobilized antigen and antibody [12,13]. Mizuta and colleagues demonstrated detection of TNT using a modified Au sensor surface immobilized with a TNT analog [14]. By incorporating an aromatic alkanedithiol and an oligo(ethylene glycol) linker they were able to achieve detection levels of 80 parts-per-trillion (ppt). Biosensors composed of fused-silica microcapillaries or resins have shown promise for the detection of TNT and RDX [15–20]. Antibodies specific for TNT or RDX immobilized on these surfaces have demonstrated recognition and specificity at the ppt to low ppb detection levels. In addition, immunoassays to detect TNT using fluorescent latex microspheres have been investigated [21,22]. Luminex^100^ based fluid array immunoassays have demonstrated pg/mL detection levels for TNT in a multiplexed assay format. This assay system which employs a two laser based flow cytometry method has the potential for discriminating up to 100 different bead sets. Although the microcapillary, SPR and other sensor formats offer excellent sensitivity, concerns arise regarding portability and fragility of sensor components when exposed to harsh environmental conditions.

As biosensors and microfluidic devices evolve so to have the platforms upon which these sensing elements reside. In an effort to design more robust, less expensive sensing platforms researchers are increasingly employing thermoplastics such as poly(methyl methacrylate) (PMMA). PMMA is inexpensive and easily adaptable to thermoforming techniques. PMMA is amenable to a variety of surface modification chemistries (acid catalyzed, plasma oxidation, UV and aminolysis) for the immobilization of biomolecules by conversion of the terminal ester groups to functional moieties (e.g., carboxylic acids or primary amines). At first glance this process may seem trivial; however, researchers have discovered that bond strength and the elasticity of PMMA can be significantly influenced by the modification chemistry employed which may invariably impact the robustness of the microchip [23,24]. A capillary immunosensor to detect the explosive, RDX using PMMA as its backbone was demonstrated [25]. Using a micro-milling technique and standard sol-gel and immunosilane chemistry provided a method of producing a microchannel for covalent attachment of antibodies specific for RDX. Levels of detection were reported as low as 10 pg/mL. In addition, an immunosensor using inline integrated microfluidic mixer grooves was developed for the detection of TNT [26]. Chevrons and stripes layered in opposite directions on the top and bottom of the PMMA microchannel were the key components of the microfluidic mixer. These unique features created a near-turbulent flow within the microchannel which resulted in improved mixing of the biomolecules generating advection patterns for increased antibody-antigen interaction. Levels of detection for TNT were reported at 50 pg/mL. Most recently, a microfluidic device containing a series of parallel high aspect ratio microchannels was replicated into PMMA from a metal mold master [27]. This allowed processing large sample volumes in the microfluidic regime where picoliter samples volumes dominate high aspect ratio microstructures. This device was used to process mL scale volumes of fluid in a matter of minutes whereby circulating tumor cells were isolated from whole blood. This application clearly demonstrates the promise of microfluidic immunosensors in the future.

This study outlines the fabrication of a multi-channeled PMMA microfluidic device that can be used as a substrate for the immobilization of monoclonal antibodies specific for the detection of TNT. Multiple microchannels (39 parallel; 25 mm channels) were hot-embossed into PMMA. The channels were coated with a sol-gel film to generate a siloxane surface for antibody immobilization. Using a displacement immunoassay format, monoclonal antibodies directed against TNT were covalently immobilized in the microchannels *via* a heterobifunctional crosslinker. AlexaFluor-cadaverine-trinitrobenzene (AlexaFluor-Cad-TNB) which served as the fluorescence reporter molecule was allowed to bind in the antibody binding sites prior to TNT exposure forming the sensing complex. In the presence of TNT, the antibody would release the fluorescence reporter molecule which would be measured with a fluorescence detector downstream. The advantage of using this design and antibody immobilization protocol is an increase in the sensing surface area and the potential for high throughput applications.

## 2. Results and Discussion

**Figure 1.**
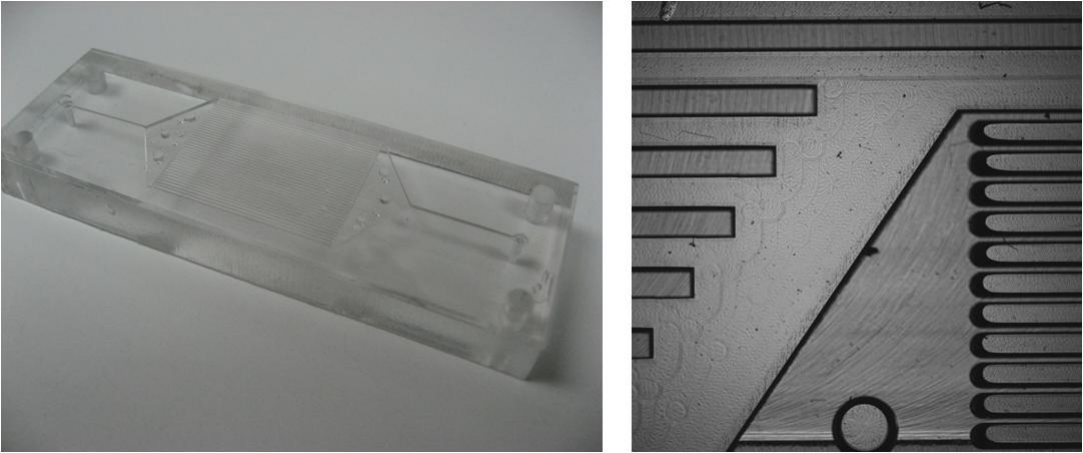
Multi-channel PMMA microfluidic device. (Left) The microfluidic device consisted of a hot-embossed 9 mm thick PMMA substrate that was tri-solvent bonded to a 1.0 mm thick PMMA coverslip. The footprint of the microfluidic device is 76 × 25 × 25 mm. The microfluidic device features flat-bottom 1.0 mm through holes at the inlet and outlet ports for fluid processing. (Right) Brightfield micrograph taken a 2x magnification of the hot-embossed substrate used to assemble the microfluidic device.

The multi-channel PMMA microfluidic device (Figure 1) was prepared *via* hot-embossing and tri-solvent annealing (see Experimental section). Microchannels were coated with a sol-gel film using a previously described protocol [25]. The sol gel film provided a means to covalently attach antibodies specific for TNT. After antibody immobilization, a fluorescent analog of TNT, AlexaFluor-Cad-TNB, was applied and allowed to bind to the immobilized antibody. Using a displacement immunoassay format, TNT was detected as low as 1 ng/mL.

**Figure 2.**
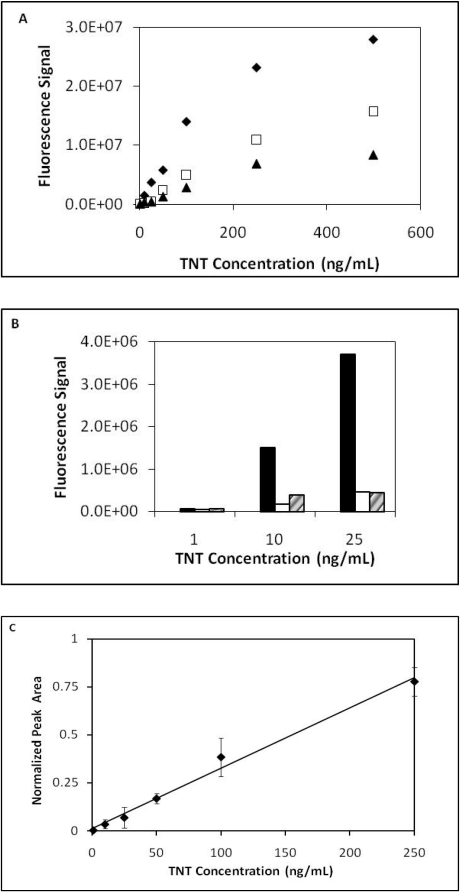
(a) Dose response curves for TNT using a multi-channel PMMA microfluidic device. Series 1 (filled diamonds), Series 2 (open squares) and Series 3 (filled triangles). (b) Bar plot of dose responses for TNT at low concentrations seen in Figure 2a. Series 1 (filled), Series 2 (open) and Series 3 (slanted). (c) Linear plot of normalized data from series 1–3 from TNT concentration 1–250 ng/mL (r^2^ = 0.989). SD ± 0.15.

TNT dose response curves from three series of sequential TNT injections (low to high concentrations) were measured and plotted (Figure 2a). From the dose response curves two observations were noted. The first observation was the continual decrease in the fluorescence signal with each successive series of TNT injections. As observed in prior work depletion in fluorescence signal over time is a common observation when using the displacement immunoassay format [18,19]. Antibody affinity, flow rate and other factors have been shown to influence the rate of fluorophore depletion [16]; however, in this instance the fluorescence response was at least 3x signal-to-noise. The second observation was the nonlinearity at the highest TNT concentration applied to the microfluidic device. This likely demonstrates we have exceeded the linear dynamic range of the assay. In addition to different degrees of binding by the antibody, antibody binding sites could be inaccessible due to the antibody orientation making displacement of the fluorescence probe more difficult. Figure 2b displays fluorescence signal responses for TNT at low concentrations (1 ng/mL, 10 ng/mL and 25 ng/mL). After normalizing the data of the three series a linear range was observed between 1 ng/mL to 250 ng/mL with an r^2^ value of 0.989 (Figure 2c).

**Figure 3.**
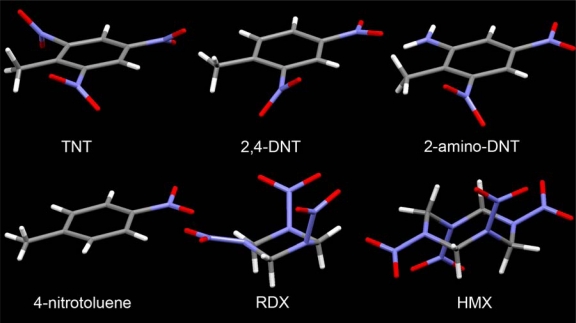
Conformation of explosives included in this study based on crystallographic studies (TNT [29], 2,4-DNT [30], 2-amino-DNT [31], 4-NT [32], RDX [33], and HMX [34]).

Further immunoassays were performed to test antibody specificity. Results showed that RDX, HMX and 4-nitrotoluene (4-NT) had minimal to no crossreactivity with the TNT antibody. Sensor response, as shown in Table 1, for the non-aromatic molecules (RDX and HMX) and 4-NT resulted in <3% crossreactivity. However, 2,4-DNT and 2-amino-4,6-DNT produced slightly higher fluorescence single responses with crossreactivity for 2,4-DNT and 2-amino-4,6-DNT at 24.8% and 14.4%, respectively. It is well known that antibody–antigen interactions can be influenced by conformation of the antigen. In this instance TNT has a nearly planar conformation with the nitro groups twisted out of the plane of the aromatic ring. This conformation is typical of nitroaromatic compounds. The non-aromatic molecules (RDX and HMX) (Figure 3) adopt very different conformations with the ring assuming a chair conformation [28] and the nitro groups in axial positions. It is not surprising that 2,4-DNT has the highest crossreactivity as its conformation is nearly identical to that of TNT while 2-amino-4,6-DNT has a lower crossreactivity possibly because of both the amino group and its effect on conformation (one of the nitro groups is in the plane of the aromatic ring).

**Figure 4.**
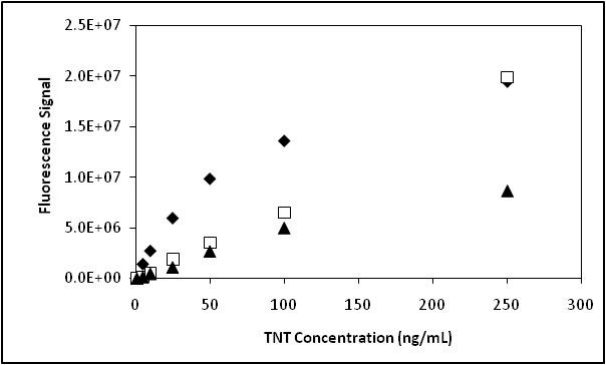
Dose response curves for TNT in seawater using multi-channel PMMA microfluidic device. TNT samples were prepared in 90% seawater: 10% flow buffer (10 mM Na-Phosphate, pH 7.4 + Tween 20 (0.01%). Series 1 (filled diamonds), Series 2 (open squares) and Series 3 (filled triangles).

To simulate environmental samples, seawater was spiked with TNT and analyzed using the modified TNT microchannel microfluidic device. In preparing the 90% seawater system flow buffer, we observed that the solution became opaque and a white precipitate began to occur. Seawater which contains a number of components; chloride, sodium, sulfate, magnesium, calcium, potassium and bicarbonates can easily form insoluble calcium phosphates when combined with sodium phosphates. To prevent the precipitate from occurring conditions were optimized by reducing the phosphate to 10 mM and the Tween 20 concentration to 0.01% for the desired system flow buffer of 90% seawater +10% flow buffer [10 mM Na Phosphate, pH7.4 + Tween 20 (0.01%)]. A transparent solution was achieved preventing any potential clogging of lines or system back pressure. TNT dose response curves from three series of sequential TNT injections (low to high concentrations) were measured and plotted (Figure 4). TNT dose response curves in seawater showed a similar trend to assays performed in PBS-Tween 20 (Figure 1) and in previously reported work [26]. A noticeable fluorescence plateau at the higher concentrations was observed.

## 3. Experimental Section

### 3.1. Antibodies, Antigens and Reagents

The monoclonal antibodies specific for TNT (mAb clone 30-1) was custom developed by Cell Essentials, Inc. - (Boston, MA, USA; http://www.cell-essentials.com/custom-monoclonalframesethtm) for NRL. 3-Mercaptopropyltrimethoxysilane, tetraethylorthosilicate (TEOS) and dimethylsulfoxide (DMSO) were obtained from Sigma-Aldrich-Fluka Corp. (Milwaukee, WI, USA). 2,4,6-Trinitrotoluene (TNT), hexahydro-1,3,5-triazine (RDX), 1,3,5,7-tetranitro-1,3,5,7-tetrazocane (HMX), 2,4-dinitrotoluene (DNT), 4-nitrotoluene (4-NT) and 2-amino-4,6-DNT were purchased from Cerilliant, Inc. (Round Rock, TX, USA). *N*-succinimidyl-4-maleimidobutyrate (GMBS) and 2,4,6-trinitrobenzene sulfonic acid (TNBSA; 5% v/v) were obtained from Pierce Chemical (Rockford, IL, USA). Seawater was obtained from Narragansett, Bay (RI, USA). AlexaFluor 647 cadaverine disodium salt was purchased from Invitrogen-Molecular Probes (Carlsbad, CA, USA). Purification of AlexaFluor-Cad-TNB was achieved using Shimadzu Prominence RP-HPLC chromatography system equipped with a Waters C18 semi-preparative column (μm Bondapak; 10 μm; 19 × 300 mm) (Milford, MA, USA) with a gradient between methanol/water/acetic acid (10/90/0.1%) and methanol/water/acetic acid (100/0/0.1%). Micro 90 was purchased from Fisher Scientific, Inc. (Pittsburgh, PA, USA). Methanol and other solvents were of HPLC grade quality grade or higher. Solvents were purchased from Mallinckrodt Baker, Inc. (Phillipsburg, NJ, USA) and Fisher Scientific, Inc.

### 3.2. High Precision Micro-Milling

A Haas Mini Mill (Oxnard, CA, USA) was used to fashion a brass molding tool out of a 6 × 101 × 50 mm brass feedstock (McMaster-Carr, Robbinsville, NJ, USA) using a 3-axis milling process. All milling bits used were obtained from Bits and Bits Co. (Silverton, OR, USA). After mounting the feed stock to the table the surface was leveled by removing 0.5 mm of the material with a 12 mm diameter dual flute end mill bit at 0.25 mm per layer. The microstructures were then machined into the plate to achieve a feature height of 0.5 mm with a combination of tapered and non-tapered flat end mill bits. The microstructures were created by removing 0.02 mm layers with each pass of the end mill bit in all subsequent cuts. The feed rate used on the x and y axes was 101 mm/min with the z-axis at 25 mm/min. In a final finishing step the features were profiled with a 0.12 mm diameter end mill bit.

### 3.3. Hot Embossing

The molding tool described above was used to replicate polymeric substrates *via* hot-embossing into poly (methyl methacrylate) (PMMA) (McMaster-Carr, Robbinsville, NJ, USA). A 101 × 101 mm brass plate was polished to a mirror-like finish and used in conjunction with an aluminum embossing chamber. The molding tool had four 6 mm diameter through holes that were used to align the embossing chamber and PMMA with the raised microarchitectures within the molding tool. With the PMMA facing up the mirrored finish of the brass plate was placed in contact with the PMMA. The embossing chamber was 6 mm thick and the PMMA feedstock was 9 mm thick which provided excess PMMA resulting in an improved mold cavity fill during the embossing stage. The entire apparatus was then situated in an embossing press (fabricated in-house) between two temperature programmable heating platens. Thermocouples were center mounted in the platens to monitor the temperature during embossing. The lower platen in the press had a fixed z-axis and the upper platen was pneumatically actuated and capable of applying 40–120 psi of downward force to the stationary platen. Without applied pneumatic pressure the heating platens were brought into conformal contact with the apparatus at room temperature, at which time a 60 psi force was applied. The temperatures of both the upper and lower platens were then programmed to achieve 170 °C. Once the final temperature was reached, the apparatus temperature was maintained for a period of 45 minutes. The apparatus was then passively cooled to 50 °C before the press was opened. The embossed substrates were removed from the molding tool by applying force to the through holes which were occupied by the alignment pegs and molded PMMA. The embossed substrates were trimmed to 76 × 25 mm using a band saw (Grizzly Industrial, Bellingham, WA, USA). Included within the microarchitecture were four additional alignment markers used to drill through holes (3 mm diameter) with a bench top mounted drill press (Ryobi, Tempe, AZ, USA). Fluidic conduits were also drilled for the influent and effluent (1 mm diameter) lines.

### 3.4. Tri-solvent Annealing

The embossed substrates were cleaned using a mild Micro 90 solution prepared by dissolving 5 mL in 1 L of de-ionized (DI) H_2_O followed by rinsing with copious amounts of reagent grade isopropyl alcohol. The substrates were placed in a Branson 2510 sonicator (Danbury, CT, USA) for 20 min. Coverslips that were 76 × 25 mm were milled from 1.5 mm thick PMMA feedstock using the Mini Mill. The coverslips were cleaned and sonicated in an analogous manner to that described above. A slight modification to a previously described tri-solvent annealing protocol [23] was implemented. Briefly, one of the coverslips was placed on a clean dry 101 × 101 × 3 mm tempered borosilicate plate and 10 drops of a solution containing 47.5% DMSO, 47.5% DI H_2_O, and 5% methanol were equally spaced on the coverslip using a Pasteur pipette. The leading edge of the embossed substrate was placed in contact with the leading edge of the coverslip. The embossed substrate was slowly lowered until conformal contact between both pieces was obtained. A second borosilicate plate was placed on top and the apparatus was clamped together with four 2 inch paper clamps (Acco Brands Inc. Lincolnshire IL, USA). The apparatus was placed in a temperature programmable oven (Agilent, Wilmington, DE, USA) and heated to 90 °C at a rate of 20 °C/min. The final temperature was maintained for 35 min before the oven was cooled to 35 °C. The devices were tested for leaks by interfacing to an Applied Biosystems 400 reciprocating pump (Foster City, CA, USA). Three aliquots of 1 mL DI H_2_O were delivered to the device at 1, 2 and 3 mL/min. The effluent was captured in tared vessels and weighed. Devices with 95–105% recovery were deemed to have no leaks and used for assays.

### 3.5. Immobilization of Antibody Specific for TNT

Monoclonal antibodies specific for TNT were immobilized within the multi-channeled PMMA microfluidic device using a previously described protocol [25]. Briefly, a sol-gel solution, tetraethylorthosilicate (TEOS), was applied to the microfluidic device *via* a syringe pump at a flow rate of 100 μL/min and allowed to incubate for 2 min. The solution was then withdrawn from the microchannel at the same flow rate leaving a sol-gel coated PMMA surface. The TEOS coated PMMA surface was allowed to incubate in a humidified oven at 60 °C for a minimum of 5h. Afterwards, the microchannels were base cleaned with 1N NaOH, rinsed with 18 MΩ Milli-Q water, acid cleaned with 5 M HCL and then treated with a 4% solution of 3-mercaptopropyltrimethoxysilane prepared in methanol for 1h at room temperature (RT). The silane-coated microchannels were rinsed with methanol and dried briefly. Next a solution of *N*-succinimidyl-4-maleimidobutyrate (GMBS; dissolved first with 2 drops of DMSO) (4 mM) in absolute ethanol was applied to the microchannels using a 1cc syringe and allowed to incubate for 1h at RT. Microchannels were subsequently rinsed with ethanol, 18 MΩ Milli-Q water then dried briefly with an N_2_ stream. Antibodies specific for TNT (1 g/L in PBS, pH 7.4) were then infused in the microchannel and allowed to incubate at 4 °C overnight. Prior to saturation of the antibody binding sites with the AlexaFluor-Cad-TNB, the microchannels were blocked with a 1% solution of bovine serum albumin (BSA) in PBS, pH 7.4 for 2 h at RT. The functionalized microchannel device containing the immobilized antibody can be stored in the liquid blocking buffer (BSA in PBS, pH 7.4) indefinitely at 4 °C until saturation with the fluorescence analog. Once the microchannel device has been saturated with the fluorescence analog (dissolved in PBS, pH 7.4) it can remain in the liquid solution until commencement of immunoassay. This allows one to process and store many microchannel devices for future immunoassays.

### 3.6. Synthesis and RP-HPLC Purification of AlexaFluor647-Cadaverine-TNB

A fluorescent analog of TNT, AlexaFluor647-cadaverine-TNB (AlexaFluor-Cad-TNB), was synthesized and purified for use in the TNT immunoassay. AlexaFluor 647 cadaverine, disodium salt (0.1 μmole) was dissolved in 0.5 mL of borate buffer saline (BBS), pH 9.0. TNBSA (10.2 μmoles; 100-fold excess) was added directly to the AlexaFluor solution and allowed to incubate with stirring for 1h at RT. Purification of AlexaFluor-Cad-TNB was achieved using Shimadzu Prominence RP-HPLC chromatography system equipped with a Waters C18 semi-preparative column (μm Bondapak; 10 μm; 19 × 300 mm) (Milford, MA, USA) with a gradient between methanol/water/acetic acid (10/90/0.1%) and methanol/water/acetic acid (100/0/0.1%). At a total flow rate of 4.0 mL/min efficient separation of the product from the initial reactant was achieved. UV/Vis detection of product was accomplished at 650 nm. The product, AlexaFluor-Cad-TNB, eluted at 30.7 min compared to the AlexaFluor 647 cadaverine which eluted at 25.0 min. The final product was dried down into amber vials and store at −20 °C until further use.

### 3.7. Displacement Immunoassay for TNT

Prior to the assay, the anti-TNT PMMA microfluidic device was filled with a 30 μM solution of the AlexaFluor-Cad-TNB dye conjugate (dissolved in PBS, pH 7.4) to saturate the antibody binding sites. Antigen binding was allowed to occur for 24 h at 4 °C. The following day excess fluorophore was removed from the microchannel using a syringe pump. The multi-channel microfluidic device was connected in-line with a Rheodyne three-way-valve HPLC injection loop (100 μL) to complete the system. Continuous fluid and sample flow throughout the system was achieved using an Applied Biosystems 400 reciprocating pump at a flow rate of 100 μL per min. Fluorescence detection was obtained with a Model FP2020-Plus Intelligent Spectrofluorometer purchased from Jasco, Inc. (Easton, MD) equipped with a 16 μL flow cell. Excitation and emission settings for the AlexaFluor-cad-TNB analog were set at 632 nm and 665 nm, respectively.

A displacement immunoassay was performed using the anti-TNT coated multi-channeled PMMA microfluidic device. TNT samples (concentrations from 0 ng/mL to 500 ng/mL) were prepared in system flow buffer (PBS, pH 7.4 + Tween 20 (0.01%) and injected into the flow stream through the HPLC injection loop. Once the TNT sample reaches the antibody binding site, the TNT will displace the fluorescence analog from the antibody pocket producing an increase in the fluorescence signal over background. Three series of TNT injections were performed with a triple rinse of the injection loop with system flow buffer between each series to minimize cross contamination. A typical immunoassay involving 3 series of TNT injections requires approximately 1.5–2.0 hours. This is based on 7–8 TNT injections per series at a flow rate of 100 μL/min that range in concentration plus additional time taken for buffer rinses between each series. Each injection is 100 μL and takes approximately 5–7 minutes from the initial injection to the peak summit then back to baseline. Total liquid volume per immunoassay can be between 9.0–12.0 mL. Using the displacement immunoassay format under continuous flow allows one the capability of increasing the flow rate, analyzing more samples and decreasing the total immunoassay time. Total fluorescence peak area for each concentration was integrated using Borwin Analytical Software that was purchased from Jasco, Inc. (Easton, MD, USA). Fluorescence dose response curves were recorded for each TNT concentration and plotted.

### 3.8. Antibody Specificity Immunoassay

To validate specificity of the TNT immunoassay, other explosives were applied to the anti-TNT multi-channel PMMA microfluidic device. Explosives with structures similar to TNT (e.g., 2,4-dinitrotoluene (DNT), 4-nitrotoluene (4-NT) and 2-amino-4,6-DNT) and explosives dissimilar in structure to TNT (e.g., RDX and HMX) were applied. Duplicate injections of each compound at a concentration of 100 ng/mL were applied to the sensor. Fluorescence signal responses were measured and recorded.

### 3.9. TNT Immunoassay in Seawater

In developing the TNT immunoassay for use in seawater a change in the flow buffer composition was required. Initial assays were performed using a system flow buffer that consisted of PBS + Tween 20 (0.01%). For the seawater assay TNT samples (concentrations from 0 ng/mL to 500 ng/mL) were prepared in the new system flow buffer composed of 90% seawater + 10% flow buffer (10 mM Na Phosphate, pH7.4 + Tween 20 (0.01%) and injected into the flow stream through the HPLC injection loop. This was performed to prevent a white precipitate from forming in the solution. Three series of TNT injections were performed. Fluorescence dose response curves were recorded for each TNT concentration and plotted.

## 4. Conclusions

The development of sensors to detect trace levels of explosives requires improvements in all aspects of the sensors’ components. Of utmost importance is immunoassay sensitivity, but key factors that must be addressed include instrument durability, reagent costs and sensor performance with complex samples containing multiple interferents. We have reported a fabrication method using PMMA for the design of a multi-channeled immunosensor to detect TNT. This method employs a hot embossing technique that produces microarchitectures/microchannels where antibodies specific for TNT can be immobilized. Using a fluorescence analog of TNT (AlexaFluor647-cadaverine-TNB) as a means of detection and the multi-channeled PMMA sensor immobilized with the TNT antibody, we were able to achieve detection levels for TNT at 1.0 ng/mL. Immunoassays conducted in seawater also provided an indication of immunoassay performance in an environmental sample. Fluorescence dose responses and detection levels were similar to those recorded in standard buffer. Crossreactivity studies showed antibody specificity for TNT compared to RDX and HMX. However, some compounds similar in structure did crossreact slightly (e.g., 2, 4-DNT and 2-amino-4, 6-DNT). 2,4-DNT and 2-amino-4,6-DNT, which are breakdown products of TNT, and can serve as a good environmental indicator of TNT contamination. Combining the design of the modified PMMA multi-channeled device and the specificity of a TNT antibody provides an inexpensive, robust sensor with the potential for high throughput real-time analysis.

## Figures and Tables

**Table 1.** Percent crossreactivity of explosives to TNT antibody. Explosive samples applied in TNT immunoassay at a concentration of 100 ng/mL. All samples were applied in duplicate. Fluorescence peak areas were averaged and percent (%) crossreactivity recorded.

**Explosive**	**Concentration (ng/mL)**	**Fluorescence Peak Area**	**% Crossreactivity**
2,4,6-Trinitrotoluene (TNT)	100	2905284	-
RDX	100	4077	1.4
HMX	100	8015	2.7
2,4- Dinitrotoluene (2,4-DNT)	100	723105	24.8
4-Nitrotoluene	100	7255	2.5
2-Amino-4,6-dinitrotoluene (2ADNT)	100	420318	14.4
